# An RDAU-NET model for lesion segmentation in breast ultrasound images

**DOI:** 10.1371/journal.pone.0221535

**Published:** 2019-08-23

**Authors:** Zhemin Zhuang, Nan Li, Alex Noel Joseph Raj, Vijayalakshmi G. V. Mahesh, Shunmin Qiu

**Affiliations:** 1 Key Laboratory of Digital Signal and Image Processing of Guangdong Province, Department of Electronic Engineering, Shantou University, Shantou, Guangdong, China; 2 Department of Electronics and Communication Engineering, BMS Institute of Technology and Management, Bengaluru, Karnataka, India; 3 Imaging Department, First Hospital of Medical College of Shantou University, Shantou, Guangdong, China; Guangdong Technion Israel Institute of Technology, CHINA

## Abstract

Breast cancer is a common gynecological disease that poses a great threat to women health due to its high malignant rate. Breast cancer screening tests are used to find any warning signs or symptoms for early detection and currently, Ultrasound screening is the preferred method for breast cancer diagnosis. The localization and segmentation of the lesions in breast ultrasound (BUS) images are helpful for clinical diagnosis of the disease. In this paper, an RDAU-NET (Residual-Dilated-Attention-Gate-UNet) model is proposed and employed to segment the tumors in BUS images. The model is based on the conventional U-Net, but the plain neural units are replaced with residual units to enhance the edge information and overcome the network performance degradation problem associated with deep networks. To increase the receptive field and acquire more characteristic information, dilated convolutions were used to process the feature maps obtained from the encoder stages. The traditional cropping and copying between the encoder-decoder pipelines were replaced by the Attention Gate modules which enhanced the learning capabilities through suppression of background information. The model, when tested with BUS images with benign and malignant tumor presented excellent segmentation results as compared to other Deep Networks. A variety of quantitative indicators including Accuracy, Dice coefficient, AUC(Area-Under-Curve), Precision, Sensitivity, Specificity, Recall, F1score and M-IOU (Mean-Intersection-Over-Union) provided performances above 80%. The experimental results illustrate that the proposed RDAU-NET model can accurately segment breast lesions when compared to other deep learning models and thus has a good prospect for clinical diagnosis.

## Introduction

Breast cancer, next to skin cancer is a disease which seriously endangers women health [1, 2]. With the development of modern medicine, if breast cancer is diagnosed early, the survival rate of patients is greatly improved. The diagnosis of a breast tumor can be divided into invasive diagnosis and non-invasive diagnosis. Invasive diagnosis, which mainly refers to biopsies, causes physical damage to the tissues, whereas non-invasive diagnosis refers to the examination of the breast lesion area, using either X-ray, MRI(Magnetic Resonance Imaging) or Ultrasound (US) imaging examination. Among various examinations, using US images, due to its low radiation, low cost and real-time output capabilities have become a preferred choice for breast tumor diagnosis.

**Fig 1:**
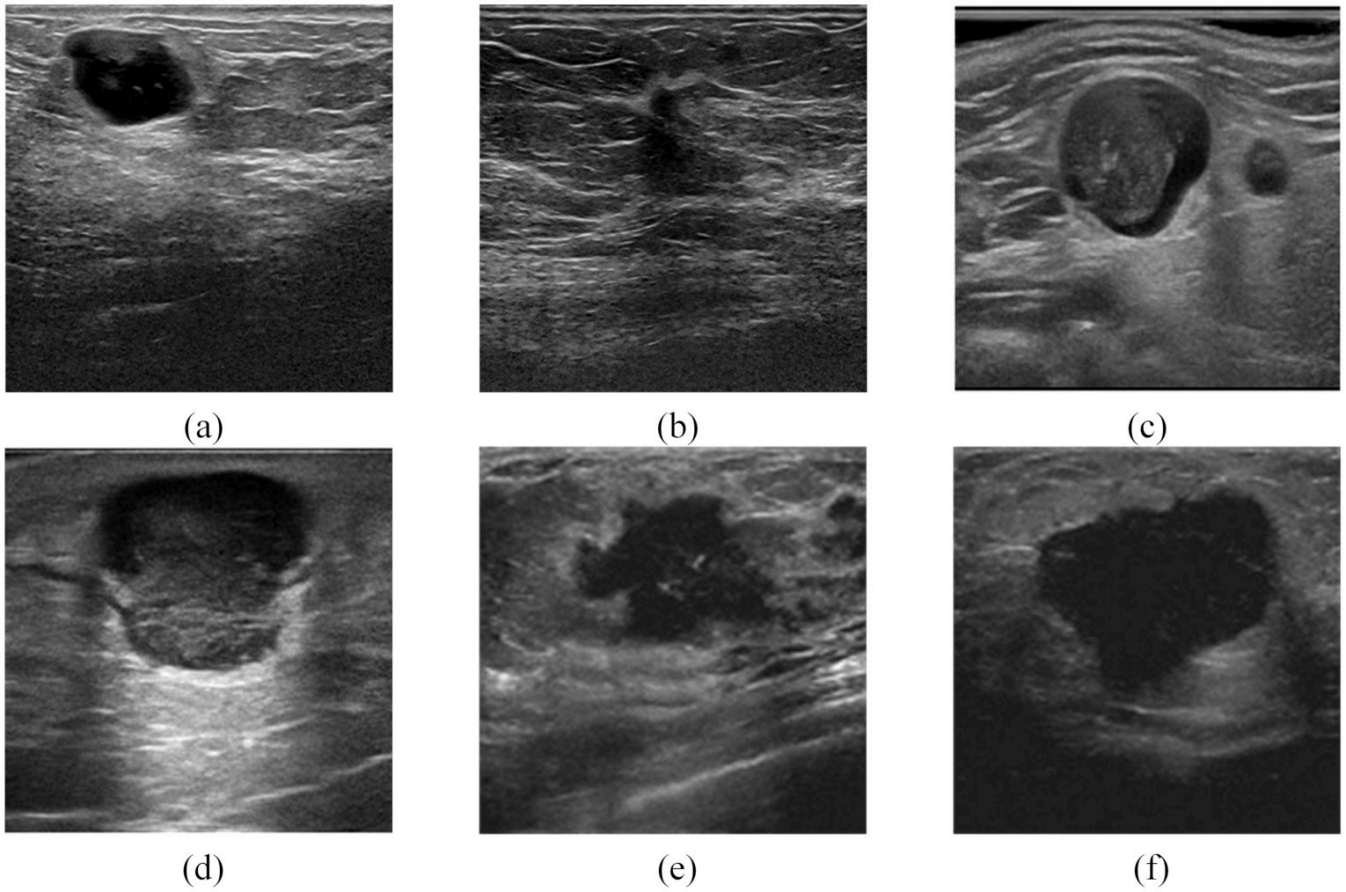
Benign and malignant tumors. (a) and (b) were obtained from Breast Ultrasound Lesions Dataset(Dataset B) [13]. (c) and (d) were acquired from Gelderse Vallei Hospital in Ede, the Netherlands [14]. (e) and (f) were obtained from the Imaging Department of the First Affiliated Hospital of Shantou University. It can be seen from these six figures that there are obvious differences between the tumor morphology and the surrounding tissues.

Image segmentation in BUS images refers to extracting the region of interest (lesion) from the normal tissue region. Fig 1 presents few BUS images with both benign and malignant tumors and it is understood that morphology of the tumor varies significantly from the surrounding tissues. This attribute forms the basis for localization and segmentation of tumors using various Machine Learning and Deep Learning techniques. The quality of the segmentation directly affects the accuracy and reliability of the diagnosis results. Due to the nature of the acquisition process, US images are affected by noise and other image artifacts’ that greatly increase the difficulty of the segmentation process. Horsch et al. [3] proposed an algorithm for BUS lesion segmentation, where the images were initially pre-processed for noise removal using a median filter. Later the processed images were intensity inverted, multiplied with Gaussian constraint function and thresholded to provide potential lesion boundaries by suppressing distant pixels. Finally, Average radial derivative function aids as a utility function to maximize the actual lesion margins. Although the threshold method is fast, parameters such as the center, height, and width were required to be provided manually for better segmentation results. Xu and Nishimura [4] proposed an algorithm for BUS segmentation using Fuzzy C-Mean (FCM) clustering which required prior initialization of a number of clusters and the noise tolerance level. These initializations were not generalized and depended on the experience, thereby affecting the overall segmentation result. Gomez et al. [5] proposed a method similar to [3] for breast ultrasound lesions segmentation. Here CLAHE and Anisotropic diffusion filter were successively used to enhance the contrast and reduce the speckle noise associated with BUS images. Then, the watershed transformation algorithm was used for finding potential lesion boundaries which were further refined by the Average radial derivative function to determine the final contour of the lesion. An overlap ratio of about 86% was reported by the authors. Daoud et al. [6] introduced a semi-automatic active contour model, which required users to provide an initialization (circular contour) within the tumor. Later, statistical parameters calculated based on the envelope signal-to-noise ratio was iteratively used to move the coordinates of the initial contour towards the tumor boundary. However, the segmentation outputs of the model largely depend on the initial contour. When the initial contour is not well positioned, the ideal segmentation outputs were not achieved. virmani et al. [7] studied the application of despeckling filtering algorithm in BUS image segmentation. The study included (a) finding the optimum number of despeckle filters from an ensemble of 42 filters and (b)evaluation of the segmentation outputs of the benign and malignant tumor. The first set of experiments provided 6 optimum filters that retained the edges and features of the image. Measures such as Beta metrics and Image Quality score were used to assess the performance of the filters. Next, the speckle-removed BUS images were segmented by the edge-based active contour model proposed by Chan and Vese [8]. The performance of the segmentation algorithm was quantitatively evaluated using Jaccard index and qualitatively by the radiologists. It was stated that the DPAD(detail preserve anisotropy diffusion) filter was able to obtain clinically acceptable images. The proposed method was tested on 104 ultrasound tumor images (43 benign and 61 malignant) and an average Jaccard index of 79.52% was reported. Daoud et al. [9] proposed a method based on super-pixels to segment the lesions in BUS images. To begin with, the BUS image was decomposed into coarse hyper-pixels to obtain the initial contour of the tumor and later the coarse pixels were refined to super-pixels to improve the final contour of the segmented tumor. The two-stage pipeline provided segmentation results which were comparable to the ground truth. Panigrahi et al. [10] proposed a novel hybrid clustering technique comprising of Multi-scale Gaussian kernel induced Fuzzy C-means (MsGKFCM) and Multi-scale Vector Field Convolution (MsVFC) to segment the region of interest within the BUS images. Initially, the BUS images were preprocessed using speckle reducing anisotropic diffusion technique [11] and then clustered as probable lesion segments using MsGKFCM. Later cluster centers were presented as inputs to MsVFC to obtain the accurate lesion boundary. The technique was tested on 127 US images and various performance measures were used to evaluate the technique. Accordingly, the average values of Jaccard index and dice similarity scores were 93.1% and 93.3% respectively. Zhuang et al. [12] proposed a fractal based technique to segment US images. Here the images of the carotid artery were enhanced using fuzzy technique and later segmented using fractal length. It was reported that fractal length based segmentation presented more accurate segmentation results than the fractal dimensions. The technique presented high qualitative values of DSC, Precision, Recall and F1 score (0.9617, 0.9629, 0.9653 and 0.9641 respectively), together with a low value of APD (1.9316).

In recent years, with the continuous development of the convolutional neural networks (CNN), semantic segmentation algorithms employing deep learning architectures have become popular. These models combine both shallow and high-level features and thus provide accurate results when compared to traditional algorithms which mainly depend on shallow features. However, the application of deep learning in medical images is still in its infancy. Xu et al. [15] proposed a method for BUS image segmentation using CNN. Volumetric (3D) mammary US images were presented to CNN to segment the US images into four major tissues: skin, fibrous gland, mass, and adipose. The idea was to treat the segmentation as a classification problem where every pixel is associated with a class label. Therefore, a large number of BUS data samples are collected and the annotated images were trained on an 8 layered CNN model comprising of convolution, pooling, fully connected and the softmax layers. To provide the classification, the softmax layer was modified to output a probability distribution array with 4 elements, whose maximum value represented one of the four class labels. An F1 score of above 80% was reported. Lian et al. [16] proposed the Attention guided U-Net model based on U-Net architecture which incorporated attention masks for accurate iris segmentation. The use of attention masks enabled the Atten-UNet to localize on the iris region instead of the whole eye. The contracting path (encoder pipeline) of Atten-UNet presented the probable iris bounding box coordinates which were then used as a mask to focus more on the iris region thereby avoiding false segmentation outputs due to the background. The model was tested on UBIRIS.v2 and CASIA-IrisV4- Distance dataset. The mean error rates achieved were 0.76% and 0.38%, respectively. Xia and Kulis [17] proposed a fully unsupervised deep learning network referred to as W-Net model. The model concatenates two U-Nets for dense prediction and reconstruction of the segmentation outputs. Also, post-processing schemes, such as fully connected Conditional Random Field and Hierarchical segmentation were successively employed to provide accurate segmentation edges and merging of over-segmented regions respectively. The model was evaluated on the Berkeley Segmentation Database (BSDS300 and BSDS500). An overlap of 60% and 59% with respect to ground truth was reported for the two datasets. Tong et al. [18] proposed a U-Net model to segment the pulmonary nodules in CT images. Initially, the pulmonary parenchyma was obtained through binary segmentation followed by the use of morphological operators. Later the segmented lung parenchyma is divided into 64x64 cubes and introduced to a modified U-Net model comprising of residual modules instead of plain neural units. The new model provided an improvement in the training speed and also prevented over-fitting. The model presented better segmentation outputs when compared to other segmentation algorithms such as Level set [19] and Graph-cut [20] techniques.

**Fig 2:**
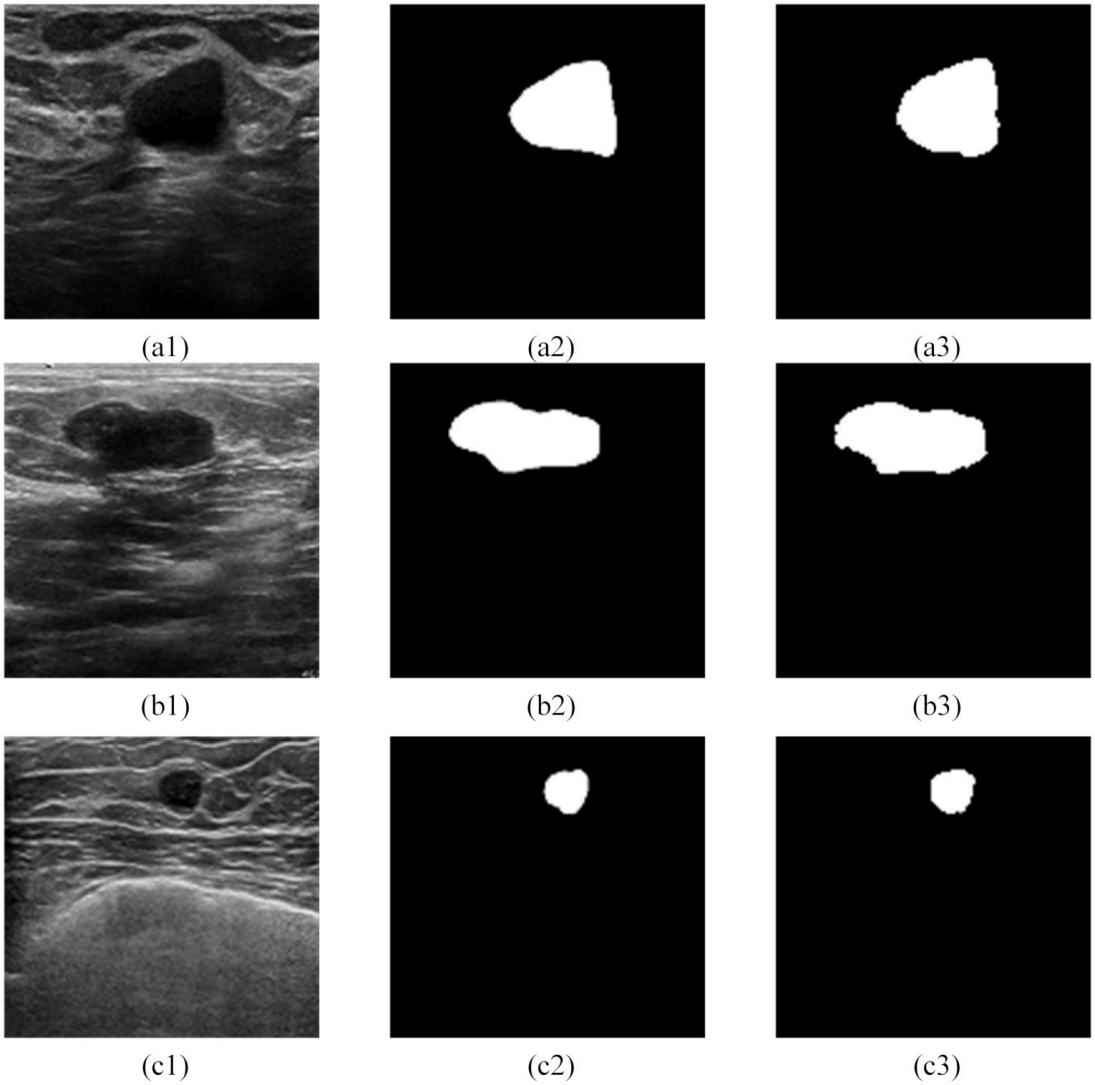
Ultrasound breast tumor segmentation based on the RDAU-NET model. Here (a1)—(c1) were obtained from Dataset B [13]. (a2), (b2), (c2) are gold standard and (a3), (b3), (c3) are the results of the RDAU-NET model segmentation.

**Fig 3:**
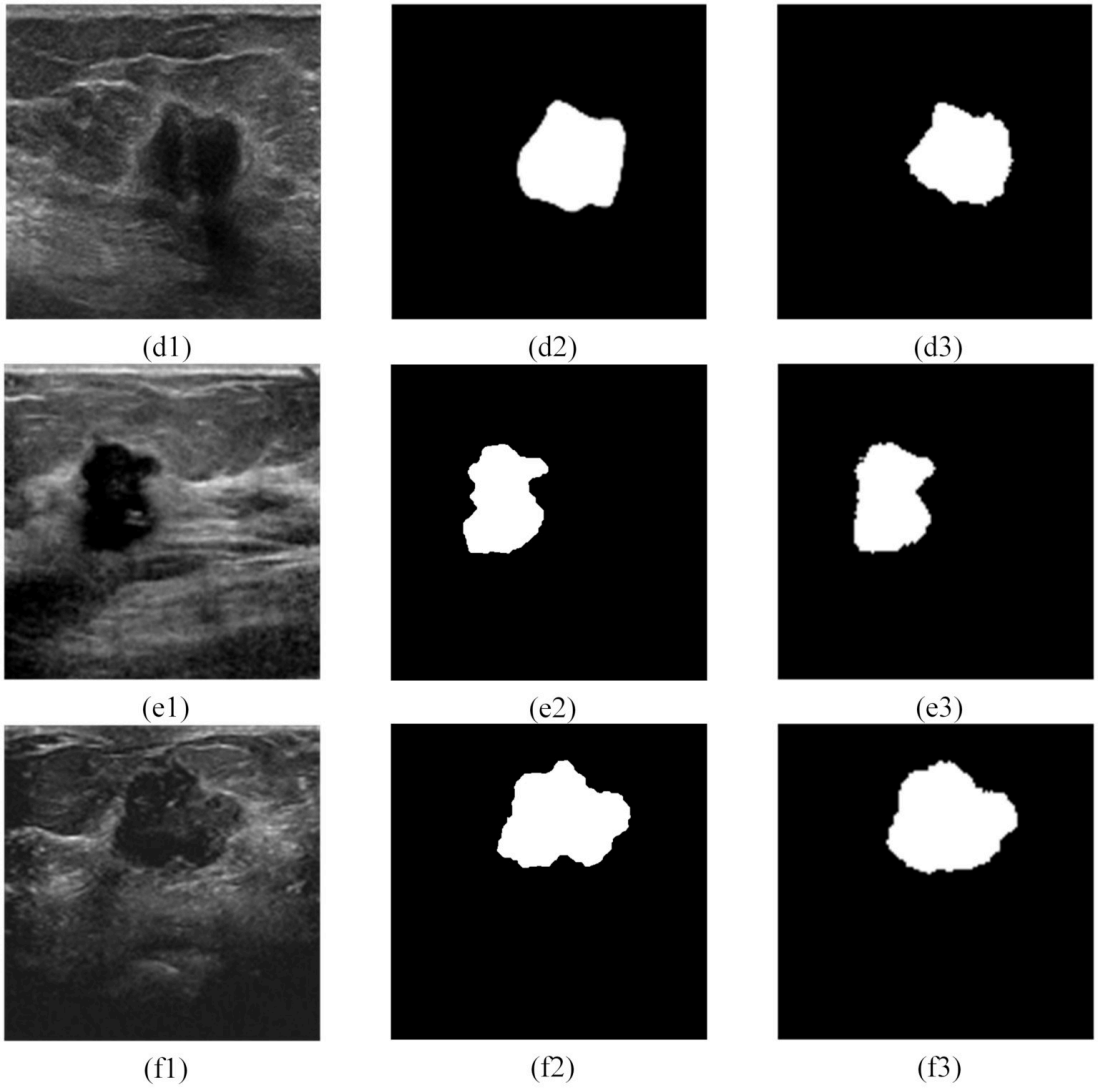
Ultrasound breast tumor segmentation based on the RDAU-NET model. Here(d1)was obtained from Dataset B [13] and (e1) and (f1) were acquired from the Imaging Department of the First Affiliated Hospital of Shantou University. Also(d2), (e2) and (f2) are gold standard and (d3), (e3), (f3) are the results of the RDAU-NET model segmentation.

Here we propose an RDAU-NET (Residual Dilated Attention Gate) architecture to segment the lesions in BUS images. Our contributions are as follows: (a) propose a model similar to [21] where residual units replace the plain neural units in the encoder-decoder structure of the U-Net structure to extract more features from the BUS image, (b) addition of dilated convolution model to the end of encoder pipeline to obtain semantic information from a large receptive field and (c) inclusion of Attention Gate(AG) system, in the skip connection part of the encoder-decoder section to suppress the irrelevant information and to effectively improve the sensitivity and prediction accuracy of the model. Figs 2 and 3 illustrates a few of the test images along with the segmentation results realized by the proposed model. The rest of the paper is organized as follows. Section 2 describes the RDAU-NET network structure, Section 3 explains the dataset and the augmentation technique adopted for training the RDAU-NET, Section 4 presents the experimental results followed by discussion and conclusion.

## Methods

### RDAU-NET network model structure

**Fig 4:**
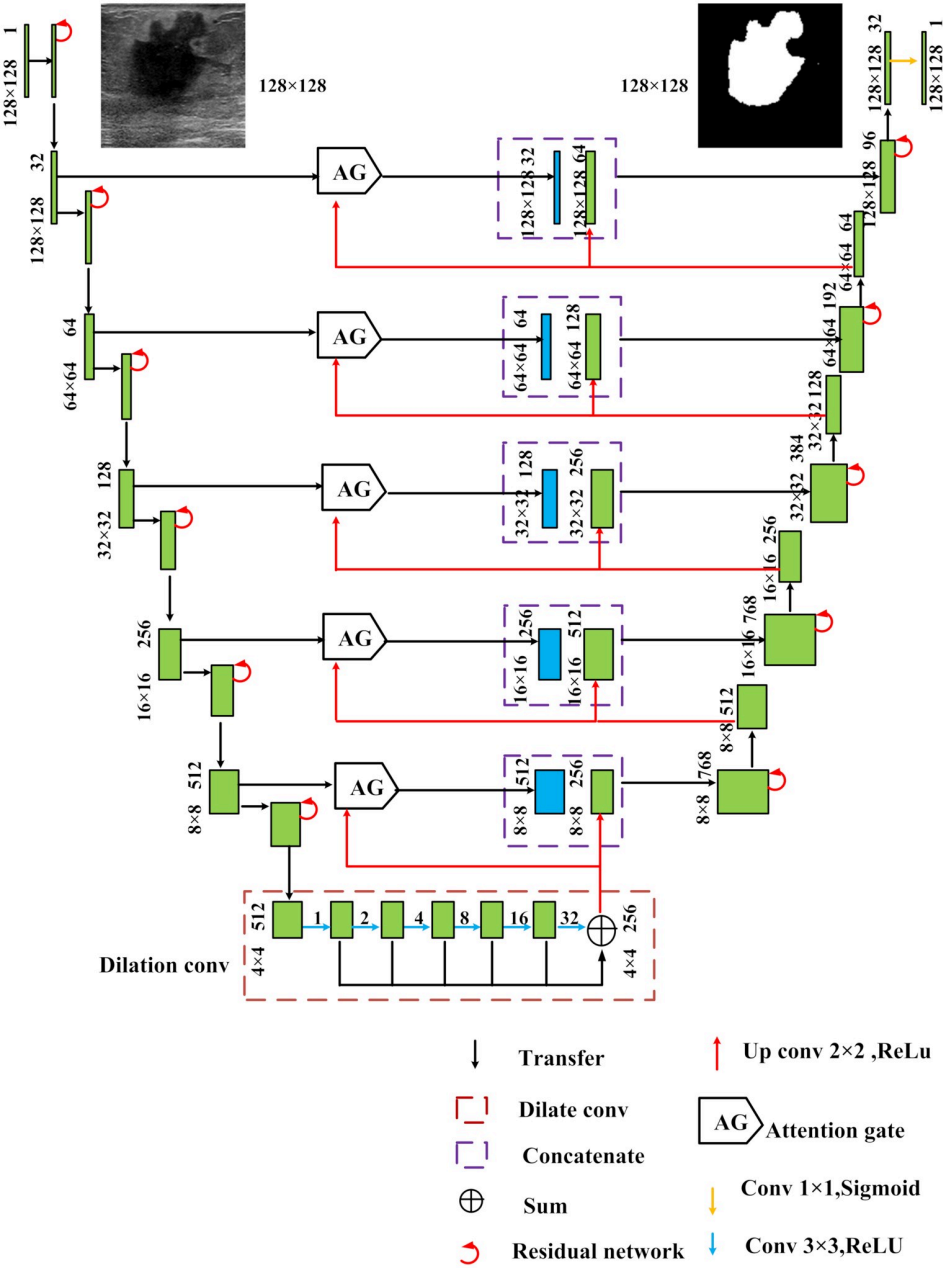
RDAU-NET model structure. The numbers above the boxes (green) indicate that the size of the input along with the number of channels. For example, 128x128 1 indicate the input resolution and the number of channels respectively. The blue box represents the outputs from Attention Gate module.

Our model is based on the U-Net architecture proposed by [22]. It has 6 residual units along the encoder pipeline which extracts the relevant features from the BUS images. Each residual unit includes a pooling operation and therefore presents a downsampled feature map at the end of the encoder pipeline. The smaller feature maps tend to reduce the accuracy of the semantic segmentation, and hence the outputs of the encoder pipeline are fed to a series of dilated convolution module with 3x3 convolution kernels and dilation ratios of 1, 2, 4, 8, 16, 32 respectively. The module outputs feature maps computed from the large receptive field which aid in improving the overall segmentation accuracy. Later, the features maps of dilated convolution module are summed and fed into a decoder pipeline consisting of 5 residual units. The decoders assist in upsampling the feature maps by concatenating the detailed feature outputs of the decoder with the corresponding high-level semantic information of the encoder. Normally traditional U-Net [22] use copying and cropping technique to facilitate the learning process, but we replace them with Attention Gate (AG) module which concentrates on learning the lesions rather than the unnecessary background. Further, the decoder pipeline restores the segmentation outputs to input image resolution and the final 1x1 convolution module presents the classification label of each pixel. Fig 4 illustrates the proposed RDAU-NET model and the following sections explain each module in detail.

### Residual network

With the increase in the number of layers, the network will have better learning ability as it progresses. However, during training, as the network starts to converge, the accuracy gets saturated and network performance degrades rapidly due to the problem referred to as the “vanishing gradients”. Therefore, we introduce residual units into the U-Net to avoid performance degradation during the training process. He et al. [23] proposed a residual learning correction scheme to avoid performance degradation which is expressed in Eq (1)

$$y=F(x,\left\{W_{i}\right\})+x$$ (1)

Here *x* and *y* are the input and output vectors of the residual block and *W*_*i*_ is the weight of the corresponding layer. The function *F*(*x*, {*W*_*i*_}) is the residual function which when added to *x* proved easier to train and learn the features than learning directly from the input *x*. Also, Eq (1) solved the “vanishing gradient” problem associated with deep networks. By taking the partial derivative of *y* with respect to *x*(Eq (2)), we can understand that the partial derivative is always greater than 1, and thus the gradient does not disappear with the increase of the number of layers.

$$\frac{\partial y}{\partial x}=1+\frac{\partial F(x,\left\{W_{i}\right\})}{\partial x}$$ (2)

Normally *F*(*x*, {*W*_*i*_}) and *x* have different dimensions and hence a correction term *W*_*s*_ is added to the input to match the dimension as shown in Eq (3).

$$y=F\left(x,\left\{W_{i}\right\}\right)+W_{s}x$$ (3)

**Fig 5:**
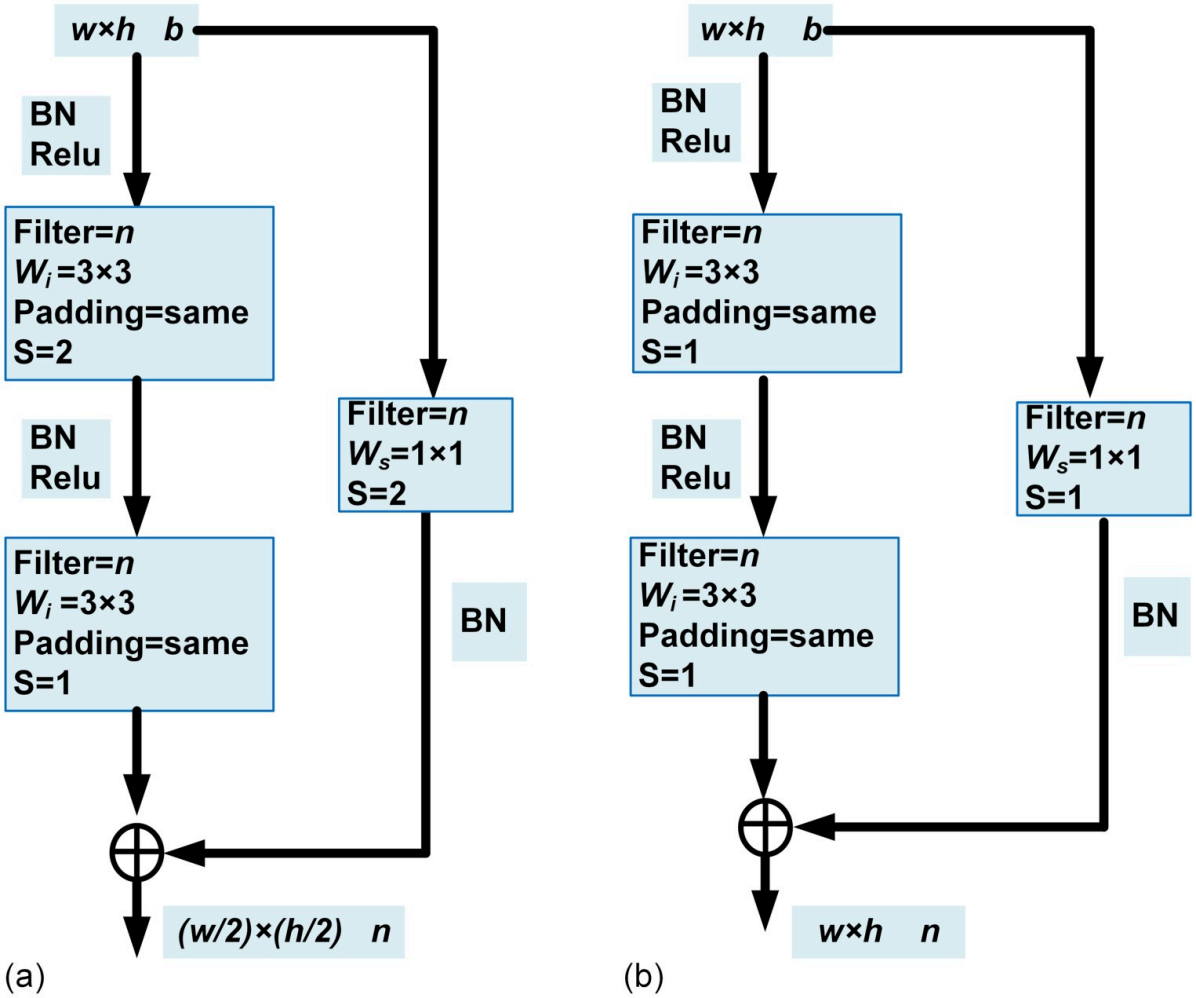
Residual units of encoder and decoder pipeline. (a) Residual units of encoder pipeline. Here *w*, *h*, and *b* represent the width, height, and channels of the input feature map, respectively. BN is batch normalization. Relu is an activation function and *n* is the number of filters. In the encoding process, the values of *n* are 64,128,256,512 and 512 for layers 2,3,4,5,6 respectively. (b)Residual units of decoder pipeline. Here values of *n* are 512, 256, 128, 64 and 32 for layers 5,4,3,2,1 respectively.

In the proposed RDAU-NET model, the inputs to the residual unit of encoder pipeline are effectively convolved with a standard 3x3 kernel and the skip connection with the 1x1 kernel(*W*_*s*_) was used to match the dimensions of the residual function. A detailed structure of the Residual unit employed in the encoder pipeline is shown in Fig 5(a). Here w x h corresponds to the width and height of the input and b represents the number of channels. Further BN, Relu, and S represent the batch normalization, activation function and the stride length (pooling operation) respectively. Also, *n* denotes the number of filtering operations performed per layer. In our model *n* takes values such as 64,128,256,512 and 512 corresponding to layers 2 to 6 of the encoder pipeline. It should be noted that the S is fixed to 2 for all the layers except for the first residual unit where it is set to 1. The decoder pipeline consists of 5 residual units which emulate the residual units of encoder section with S = ‘1’ to allow the input and output to have the same resolution. Fig 5(b) illustrates the residual unit of the decoder pipeline. The proposed RDAU-NET model avoids performance degradation issues and greatly reduces the difficulty involved in training a deep network. Further, it effectively improves the feature learning ability and is beneficial for the extraction of complex feature patterns of BUS images, thus improving the segmentation results significantly.

### Dilation convolution

In CNN architectures, due to convolution and pooling operations, the network present feature maps with less spatial information that affects the overall segmentation accuracy. Since the encoder pipeline of the U-Net represents an FC-CNN (Fully Connected CNN), dilated convolution modules are often employed in U-Nets [24, 25] to improve the receptive field. Eq (4) illustrates the dilated convolution operation between the input image *f(x,y)* and kernel *g(i,j)*.

$$z\left(x,y\right)=\sigma \{\sum_{i,j}f\left(x+i\times r,y+j\times r\right)\times g\left(i,j\right)+\beta \}$$ (4)

Here *σ* is Relu function, *β* is a biased unit, and *r* represents the dilation parameter that controls the size of receptive fields. In general, the size of the receptive field can be expressed as:

$$N={\left\{\left(K_{size}+1\right)\times \left(r-1\right)+K_{size}\right\}}^{2}$$ (5)

**Fig 6:**
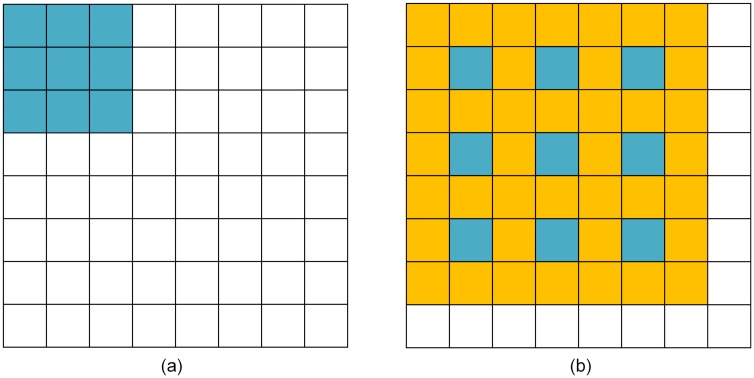
Illustration of receptive fields for r = 1 and r = 2. (a) and (b) illustrate the visual field of a 3x3 convolution kernel with *r* = 1 and *r* = 2 respectively. When *r* = 2, though the kernel parameters remain the same, the receptive field has increased to 7x7 (shown as the orange and blue parts in(b)) when compared to traditional convolution (*r* = 1, as shown in the blue part of (a)). Therefore the dilation process increases the size of the receptive field and compensates for the subsampling.

Where *K*_*size*_ is the size of the convolution kernel, *r* is the dilation parameter, and *N* is the size of the receptive field, which is illustrated in Fig 6.

**Fig 7:**
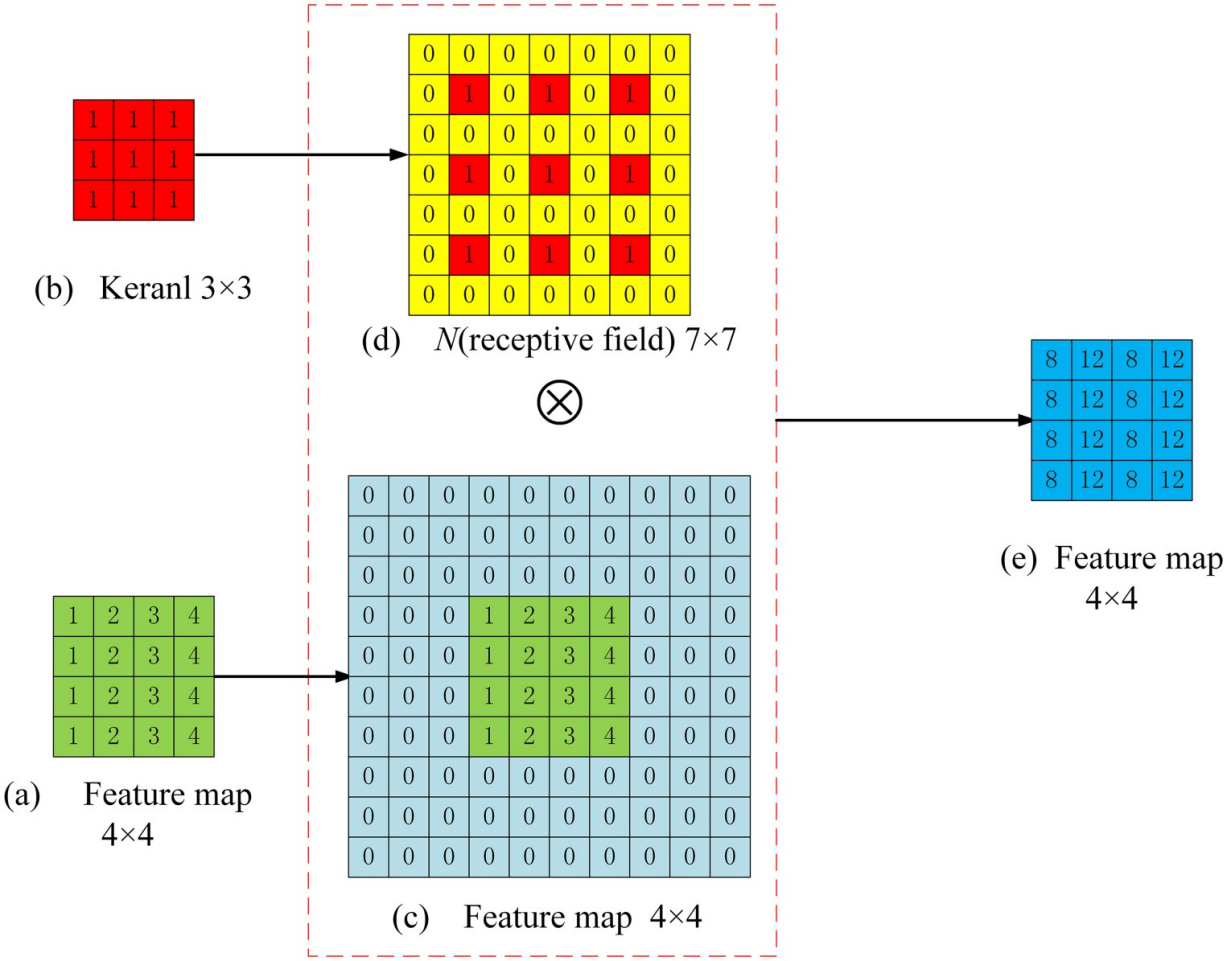
Illustration of the dilated convolution module. Here the dilation parameter r = 2, the stride size S = 1, the input feature map is 4x4, kernel size is 3x3 and receptive filed *N* is 7x7. After processing using dilated convolution, the size of the original feature map remains the same but the receptive fields increases while keeping the parameters of the model intact.

In the RDAU-NET model the feature maps of size 4x4 obtained at the end of the encoder pipeline are fed into a series of dilated convolution modules with *r* = 1,2,4,8,16,32 and *N* = 3x3, 7x7, 15x15, 31x31, 63x63 and 127x127 respectively and the outputs of the six convolutions are added, upsampled (by a factor of 2) and then fed into the decoder pipeline as shown in Fig 4. It should be noted that in the dilation convolution module, output feature maps have the same size as that of inputs but contain information from a wide range of receptive fields which greatly improves the feature learning ability of the network as illustrated in Fig 7.

### Attention Gate (AG) module

Although dilated convolutions improve the feature learning ability of the network, still there are difficulties in reducing the false predictions of small objects that have large shape variations [26]. This is mainly due to the loss of spatial information in the features maps obtained at the end of the encoder pipeline. In order to improve the accuracy, the existing segmentation framework schemes [27–29] rely on the addition of object positioning models to simplify the task obtaining the spatial attributes. Oktay et al. [26] proposed the attention U-Net network, which integrated the AG module into the U-Net model to realize spatial localization and subsequent segmentation. The AG module eliminated the need for training multiple models which required a large number of additional training parameters. In addition, compared to the positioning model used in multi-level U-Net network, the AG module gradually suppresses the feature response in the irrelevant background regions and strengthens the learning ability of foreground [30].

**Fig 8:**
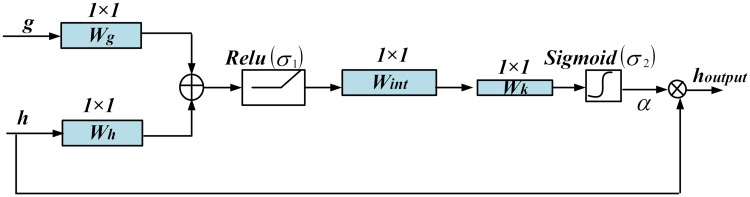
Schematic diagram of the Attention Gate (AG).

The AG model derives attention coefficients that aid in improving the segmentation accuracy. Here the coefficients are computed by combining “rich feature maps with low spatial information” obtained from the upsampled decoder layers with the high-level semantic outputs of the corresponding encoder layer. Once the gating coefficients are computed, they are element-wise multiplied with the encoder output to retain the significant activation [31]. The structure of the AG module is shown in Fig 8 and the attention coefficients are computed as based in Eqs (6) and (7)

$$\alpha =\sigma_{2}\left\{W_{k}\left[W_{int}\left(\sigma_{1}\left(W_{h}\times h+W_{g}\times g+b_{h,g}\right)\right)+b_{int}\right]+b_{k}\right\}$$ (6)

$$\sigma_{2}\left(x\right)=\frac{1}{1+exp(-x)}$$ (7)

Here *α* ∈ [0, 1] denote the computed attention coefficients, *g* and *h* represent the feature maps presented to the inputs of AG module from the decoder and encoder pipelines respectively and *W*_*g*_, *W*_*h*_, *W*_*int*_, *W*_*k*_ indicate the convolution kernels. We choose the kernel size as 1x1 to reduce the number of training parameters and the computational complexity. Also *σ*_2_ is sigmoid activation function which limits the range between 0 and 1 and *σ*_1_ is the Relu function. Here sigmoid was chosen over softmax since it provided dense activations at the output [26]. AG module outputs the constructive features through elementwise multiplication of *α* with the corresponding encoder layer output as given by Eq (8).

$$h_{output}=\alpha \times h$$ (8)

The output of the AG module (*h*_*output*_) filters out the irrelevant context information and aggravate the useful feature information that effectively improves the sensitivity and prediction accuracy of the model. Further, when compared to [31], since the *h* and *g* are of the same resolution, our AG module eliminates the need for the computationally intensive interpolation operation and thus operate faster with less memory requirement.

## Materials

### Data collection

This study considered a total of 1062 BUS images obtained from three different sources: (a) GelderseVallei Hospital in Ede, the Netherlands [14], (b) First Affiliated Hospital of Shantou University, Guangdong Province, China, and (c) BUS images obtained from Breast Ultrasound Lesions Dataset (Dataset B) [13]. The performance evaluation was based on cross-validation where the training set was used to train the proposed RDAU-NET model and the validation set was considered for fine-tuning the parameters. The optimized model was tested for segmentation performance and generalization ability using the samples of the testing set. For training and validation, we used the BUS images from [14]. The training and validation set contained 730 and 127 samples respectively. The test set consisted of 205 samples comprised of 163 BUS images obtained from Dataset B [13]and 42 BUS images provided by the Imaging Department of the First Affiliated Hospital of Shantou University. The BUS images obtained from Shantou First Affiliated Hospital were acquired using the GE Voluson E10 Ultrasound Diagnostic System(L11-5 50mm broadband linear array transducer, 7.5MHz frequency) The training, validation and the test images contained both malignant and benign BUS lesions. The RDAU-NET structure proposed in the work uses Keras (2.1.6) framework and calls Tensorflow (1.11.0). The entire model was executed using GPU TITAN XP with operating system Ubuntu version 14.04, CUDA version 9.0, cuDNN version of 7.1.2 and graphics card’s memory of 12GB.

### Data processing

**Fig 9:**
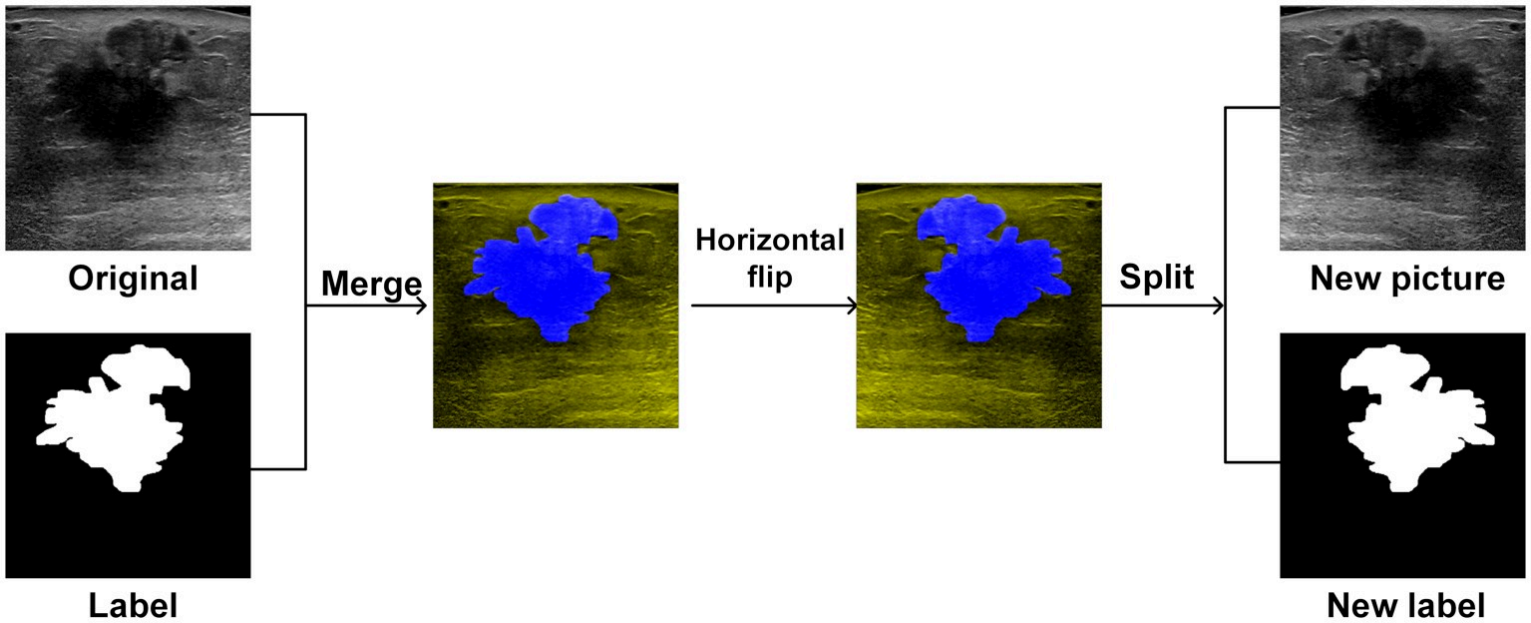
Data augmentation illustrating horizontal flipping to expand the training dataset.

To accomplish the task of cross-validation on the dataset, the training dataset had to be labeled. The BUS images from [14] were manually segmented and labeled(ground truth) by the specialist with more than 7 years of experience at the First Affiliated Hospital of Shantou University. To achieve a good segmentation under the limited number of training samples, data augmentation [32] was performed to expand the training data set. Here we first merge the BUS images and their ground truths together and then perform four affine transformations(shift along the vertical axis, shift along the horizontal axis, shear transformation and flipping about the horizontal plane) to obtain a new transformed image. Later the transformed image and its new ground truth are separated and appended to the training set as additional training images. Thus 730 images of the training set were expanded to obtain 2919 images. The data augmentation process is pictorially illustrated in Fig 9.

## Results and discussion

Two separate experiments were performed to illustrate the effectiveness of the proposed RDAU-NET model: (a) The best input image resolution that can provide a good qualitative and quantitative segmentation results when used with RDAU-NET model (b) Performance evaluation of the segmentation outputs of RDAU-NET model over FCN8s, FCN16s [33], U-Net [22], SegNet [34], Residual U-Net [35], Squeeze U-Net [36], Dilated U-Net [37], RAU-NET (Residual-Attention-UNet), DAU-NET(Dilated-Attention-UNet), and RDU-NET(Residual-dilated-UNet), The performance of the segmentation outputs was evaluated using the 9 evaluation indices: Accuracy(Acc), Precision(Pc), Recall, Dice coefficient(DC), Mean-Intersection-Over-Union(M-IOU), Area-Under-Curve(AUC), Sensitivity(Sen), Specificity(Sp) and F1score(F1). These performance indicators were computed as follows:

1. Dice coefficient [38]: It represents the degree of similarity between the segmented output of the proposed model and the gold standard. The higher the similarity between the tumor region and the gold standard, the greater is the Dice coefficient and better the segmentation result. The Dice coefficient is calculated as,
  $$Dice\;Coefficient\left(DC\right)=\frac{2\times (X\cap Y)}{\left(X+Y\right)}$$ (9)
  Also, the dice coefficient loss (Dice_loss) is the loss and is computed as follows.
  $$Dice\;Coefficient\;Loss\left(Loss\right)=1.0-\frac{2\times (X\cap Y)}{\left(X+Y\right)}$$ (10)
  where, *X* is the gold standard, which is the average result marked by two clinical experts, *Y* is the tumor area segmented by the model and *X* ∩ *Y* represents the area of overlap between the gold standard and the segmented output of the model.
2. Mean-Intersection-over-Union(M-IOU) [39]: is defined as the average ratio between the intersection and union of the gold standard and the segmented output of the model. It provides a measures coincidence between the gold standard and the segmented output of proposed the model. Higher the coincidence, greater is the M-IOU and better is the segmentation accuracy M-IOU is expressed as follows. Where *N* is the number of IOU.
  $$IOU=\frac{X\cap Y}{X\cup Y}$$ (11)
  $$M\;-\;IOU=\frac{\sum_{i=1}^{N}IOU_{i}}{N}$$ (12)
3. Performance indicators that are obtained from the confusion matrix: The Accuracy, Precision, Sensitivity, Specificity, and F1 score. These are associated with true positive (TP), true negative (TN), false positive (FP) and false negative (FN) of the confusion matrix. Here we have explained them in the Table 1. TP, FP, FN and TN are the numbers of pixels corresponding to the four categories and the formula of performance indicators is shown in Table 2.
4. The area under the curve (AUC): AUC is the area under the receiver operating characteristic (ROC) curve. It represents the degree or the measure of separability and indicates the capability of the model in distinguishing the classes. Higher the AUC better is the segmentation output and hence the model.

**Table 1:** Definition of the abbreviations.

Category	Actual lesion	Actual non-lesion
Predicted lesion	True Positive(TP)	False Positive(FP)
Predicted non-lesion	False Negative(FN)	True Negative(TN)

**Table 2:** The formula of performance measure.

Performance Measure	Formula	Description
Accuracy(Acc)	$\frac{TP+TN}{TP+FP+FN+TN}$	A ratio of the number of correctly predicated pixels to the total number of pixels in the image
Precision(Pc)	$\frac{TP}{TP+FP}$	A ratio of the number of correctly predicated lesion pixels to the total number of predicted lesion pixels
Recall/sensitivity(Sen)	$\frac{TP}{TP+FN}$	A ratio of the number of correctly predicated lesion pixels to the total number of actual lesion pixels
F1score(F1)	$2\times \frac{Precision\times Sensitivity}{Precision+Sensitivity}$	A measure for the accuracy
Specificity(Sp)	$\frac{TN}{TN+FP}$	A ratio of the number of correctly predicated non-lesion pixels to the total number of actual non-lesion pixels

### Qualitative and quantitative analysis of the RDAU-NET model for different input image resolutions

**Fig 10:**
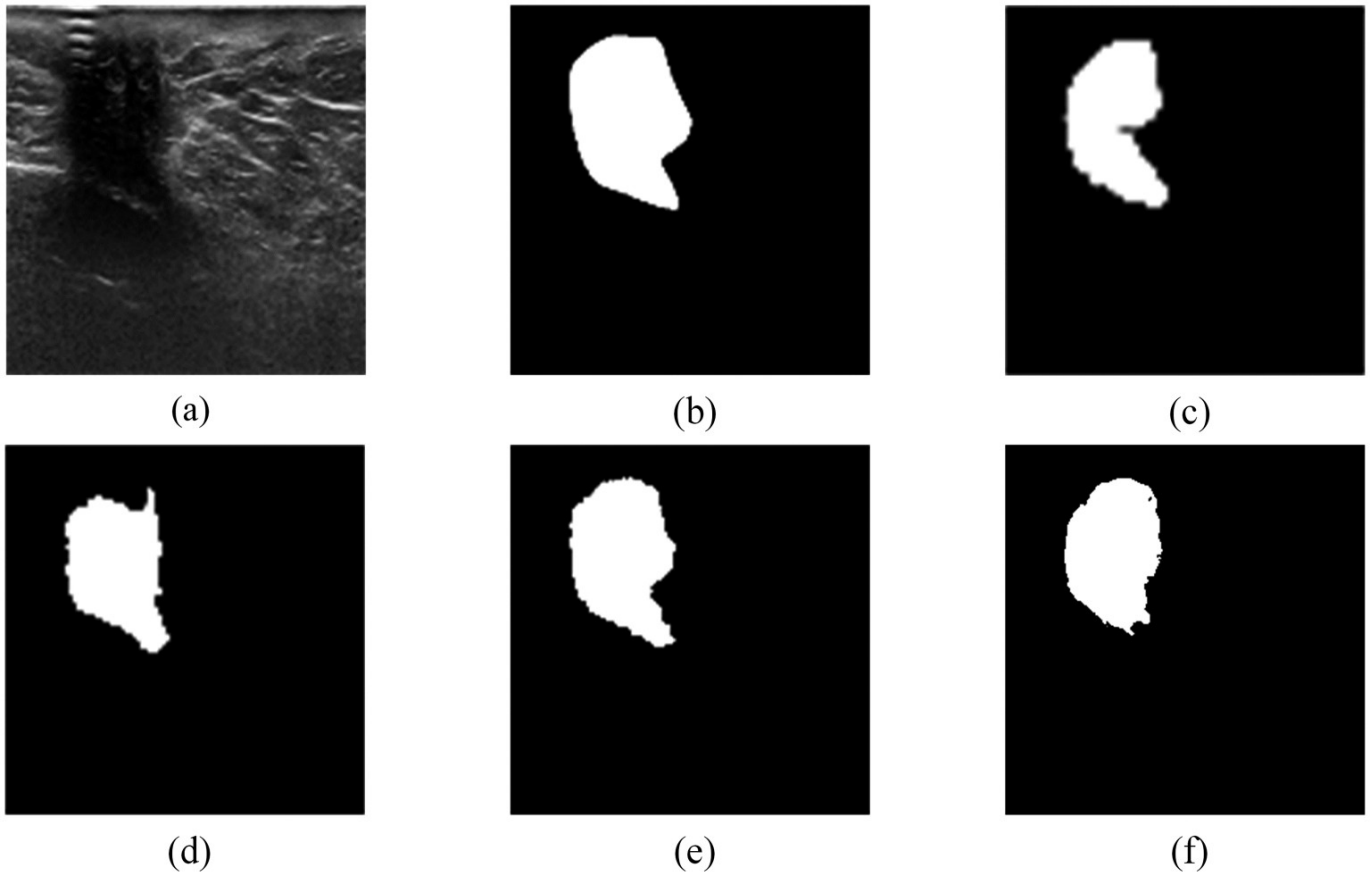
Sample image from Dataset B [13]. (a) image of malignant invasive ductal carcinoma. (b) Gold standard. (c-f) are the results of segmentation for input sizes are 64x64, 96x96, 128x128, and 256x256 respectively.

As a preliminary experiment, the segmentation task on BUS images was performed with 4 different network input sizes of 64x64, 96x96, 128x128 and 256x256 pixels. During the experiment, the number of training epochs was set to 300 and the batch size for 64x64, 96x96, 128x128 was selected as 32 while the batch size of 256x256 was 16. The batch sizes were mainly chosen to reduce the computational overhead and satisfy the memory requirements. The segmentation results of the experiment are shown in Fig 10 and Table 3 illustrates the performance metrics computed using Eqs (9) to (12) and Table 2. From Fig 10(a) to Fig 10(f), it can be seen intuitively that the best automatic segmentation results were obtained for the input image size of 128x128 pixels (Fig 10(e)). Also, the performance metrics (Table 3) emphasize that the maximum values are obtained for the input size of 128x128 pixels. In terms of computation time, though the inputs of size 64x64 pixels presented the least time, their segmentation results were not accurate. Therefore, the experiments were on focused on using 128x128 as the input image resolution for further evaluations and comparisons.

**Table 3:** Quantitative evaluation of BUS images of different input sizes.

Input Size	Loss	Acc	DC	Sen	Sp	F1	Pc	M-IOU	AUC	Train(min)
64 × 64	0.2033	0.9758	0.7966	0.7921	0.9914	0.7968	0.8471	0.7863	0.9094	75
96 × 96	0.1713	0.9775	0.8286	0.8232	0.9920	0.8291	0.8669	0.8019	0.9186	105
128 × 128	0.1530	0.9791	0.8469	0.8319	0.9934	0.8478	0.8858	0.8067	0.9227	140
256 × 256	0.1664	0.9668	0.8335	0.8208	0.9935	0.8403	0.8807	0.7992	0.9147	435

### Performance evaluation of the segmentation outputs of the RDAU-NET with other models

#### Qualitative comparison with other models

**Fig 11:**
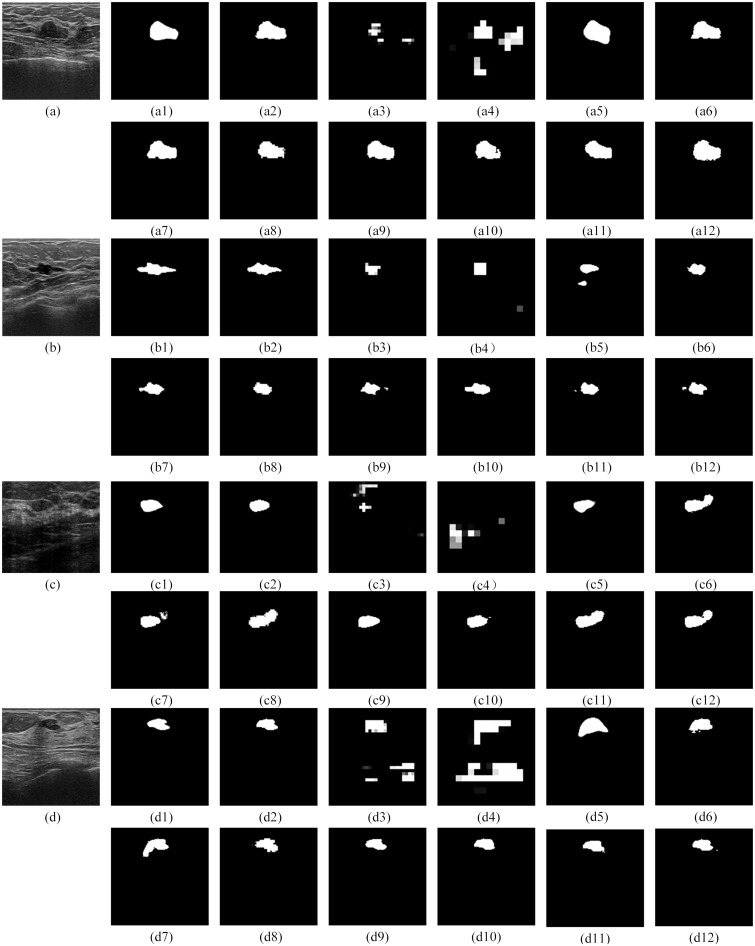
Segmentation outputs for the BUS images from the test dataset. The test dataset was obtained from Dataset B. Fig 11(a—d) illustrate the results for test images obtained from Dataset B. (a1), (b1), (c1), (d1) are the gold standard. (a2)—(a12), (b2)—(b12), (c2)—(c12), (d2)—(d12) are the segmentation results from RDAU-NET, FCN8s, FCN16s, SegNet, U-Net, Residual U-Net, Squeeze U-Net, Dilated U-Net, RAU-NET, DAU-NET, RDU-NET respectively.

**Fig 12:**
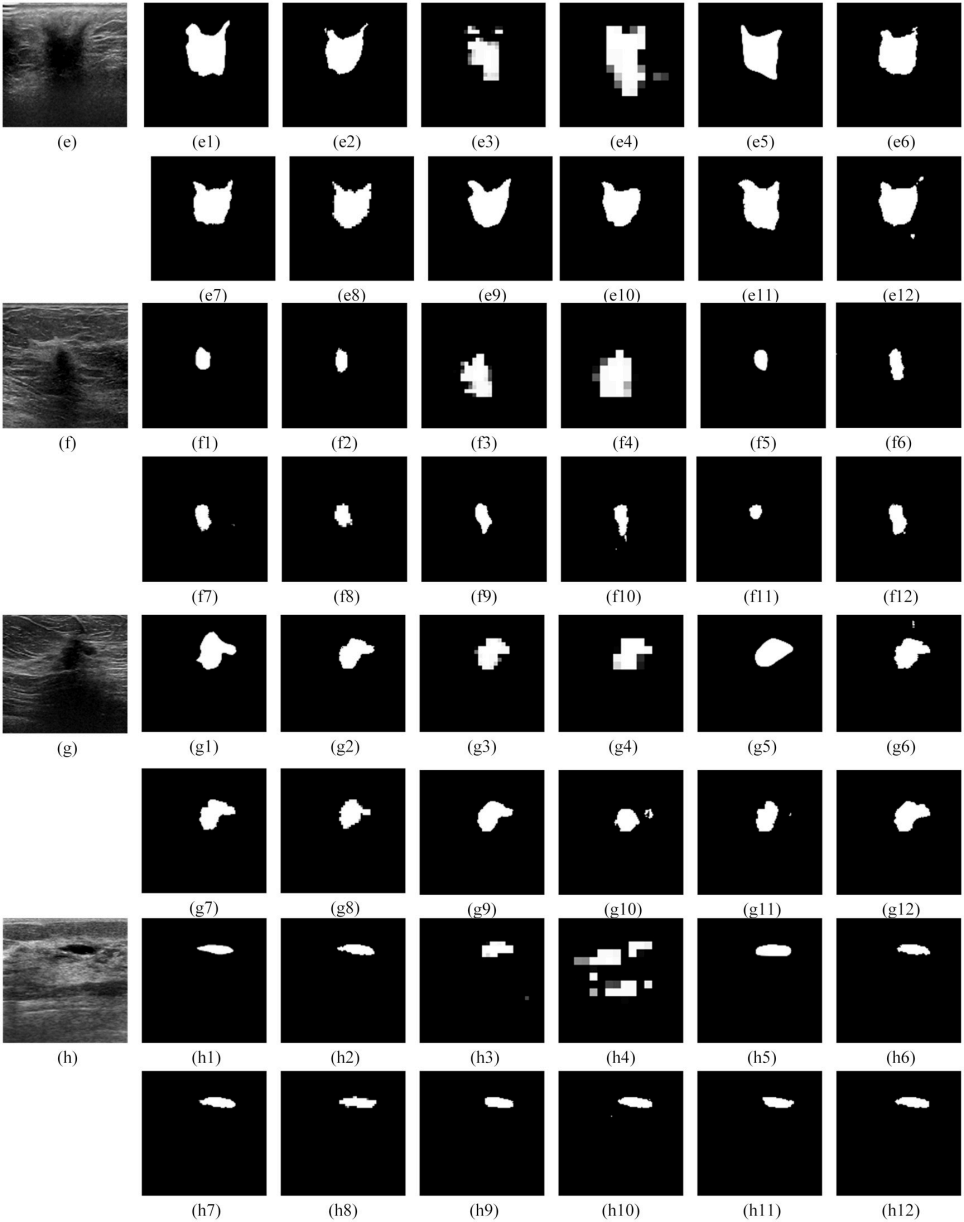
Segmentation outputs for the BUS images from the test dataset. The test dataset was obtained from Dataset B. Fig 12(e—h) illustrate the results for test images obtained from Dataset B. (e1), (f1), (g1), (h1) are the gold standard. (e2)—(e12), (f2)—(f12), (g2)—(g12), (h2)—(h12) are the segmentation results from RDAU-NET, FCN8s, FCN16s, SegNet, U-Net, Residual U-Net, Squeeze U-Net, Dilated U-Net, RAU-NET, DAU-NET, RDU-NET respectively.

**Fig 13:**
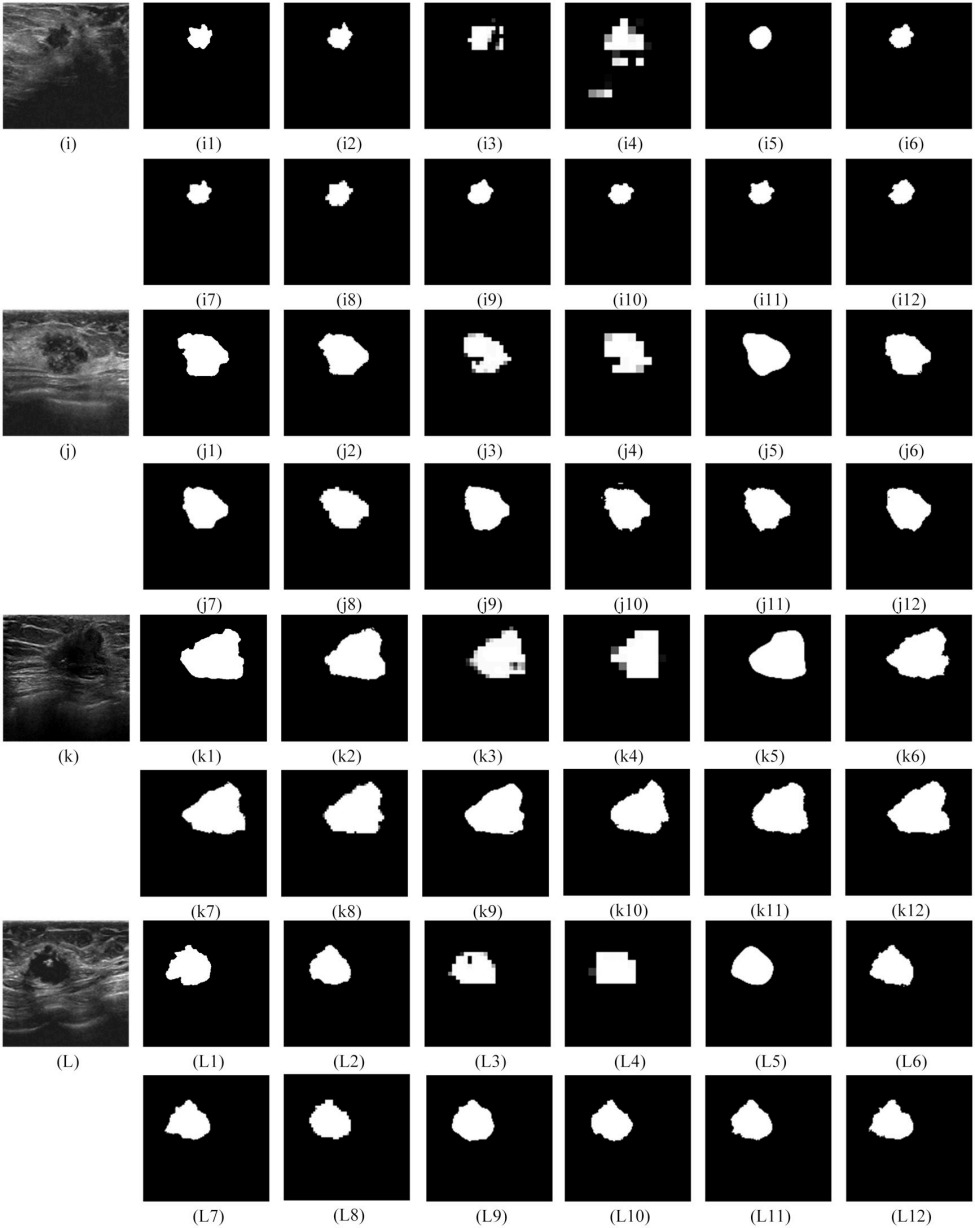
Segmentation outputs for the BUS images from the test dataset. The test dataset was obtained from the Imaging Department of the First Affiliated Hospital of Shantou University. Fig 13(i—L) represents the outputs for the test images acquired from Imaging Department of the First Affiliated Hospital of Shantou University. (i1), (j1), (k1) and (L1) are the gold standard. (i2)—(i12), (j2)—(j12), (k2)—(k12) and (L2)—(L12) are the segmentation results from RDAU-NET, FCN8s, FCN16s, SegNet, U-Net, Residual U-Net, Squeeze U-Net, Dilated U-Net, RAU-NET, DAU-NET, RDU-NET respectively.

For the qualitative performance comparison, the segmentation results of FCN8s, FCN16s, SegNet, U-Net, Residual U-Net, Squeeze U-Net, Dilated U-Net, RAU-NET, DAU-NET, RDU-NET, and RDAU-NET models are presented in Figs 11–13. In all these cases the input images that were tested were of size 128x128 and segmented outputs are of the same size.

The qualitative comparison presents the following conclusions:

1. The segmentation results of FCN8s and FCN16s are rough, with details being neglected, especially at the edges which show jagged contours leading to poor segmentation outputs.
2. Squeeze U-Net, RAU-NET, DAU-NET, RDU-NET present segmentation outputs better than SegNet and U-Net models.
3. The RDAU-NET model presents visually better segmentation results than other models and the final segmentation outputs are close to the gold standards. Also, the segmentation outputs of RDAU-NET model are superior when compared to Residual U-Net and Dilated U-Net.

**Fig 14:**
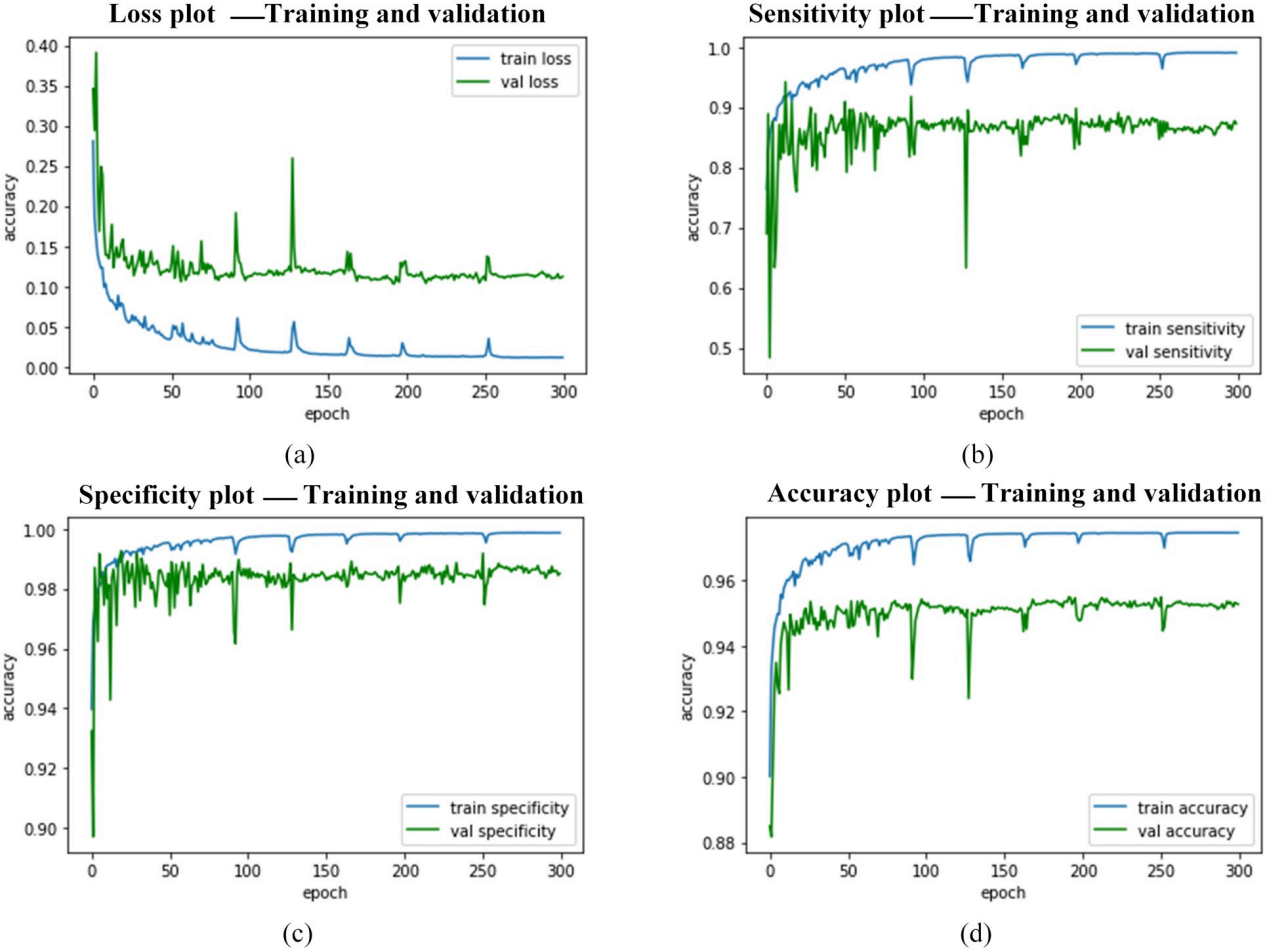
RDAU-NET performance indicators for training, validation. Here the plots (a—d) represent the performance metrics during training and validation.

**Fig 15:**
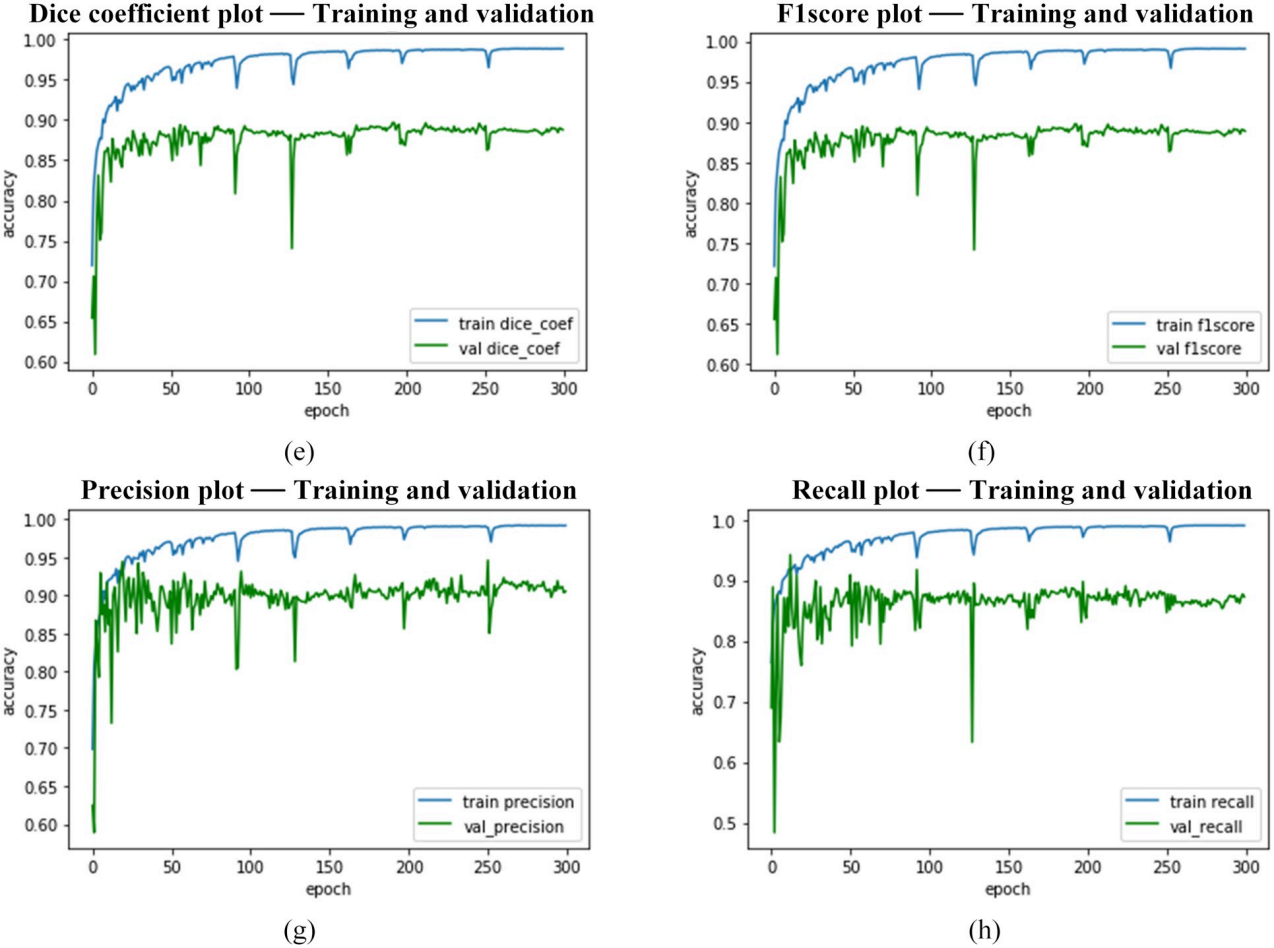
RDAU-NET performance indicators for training, validation. Here the plots (e—h) represent the performance metrics during training and validation.

**Fig 16:**
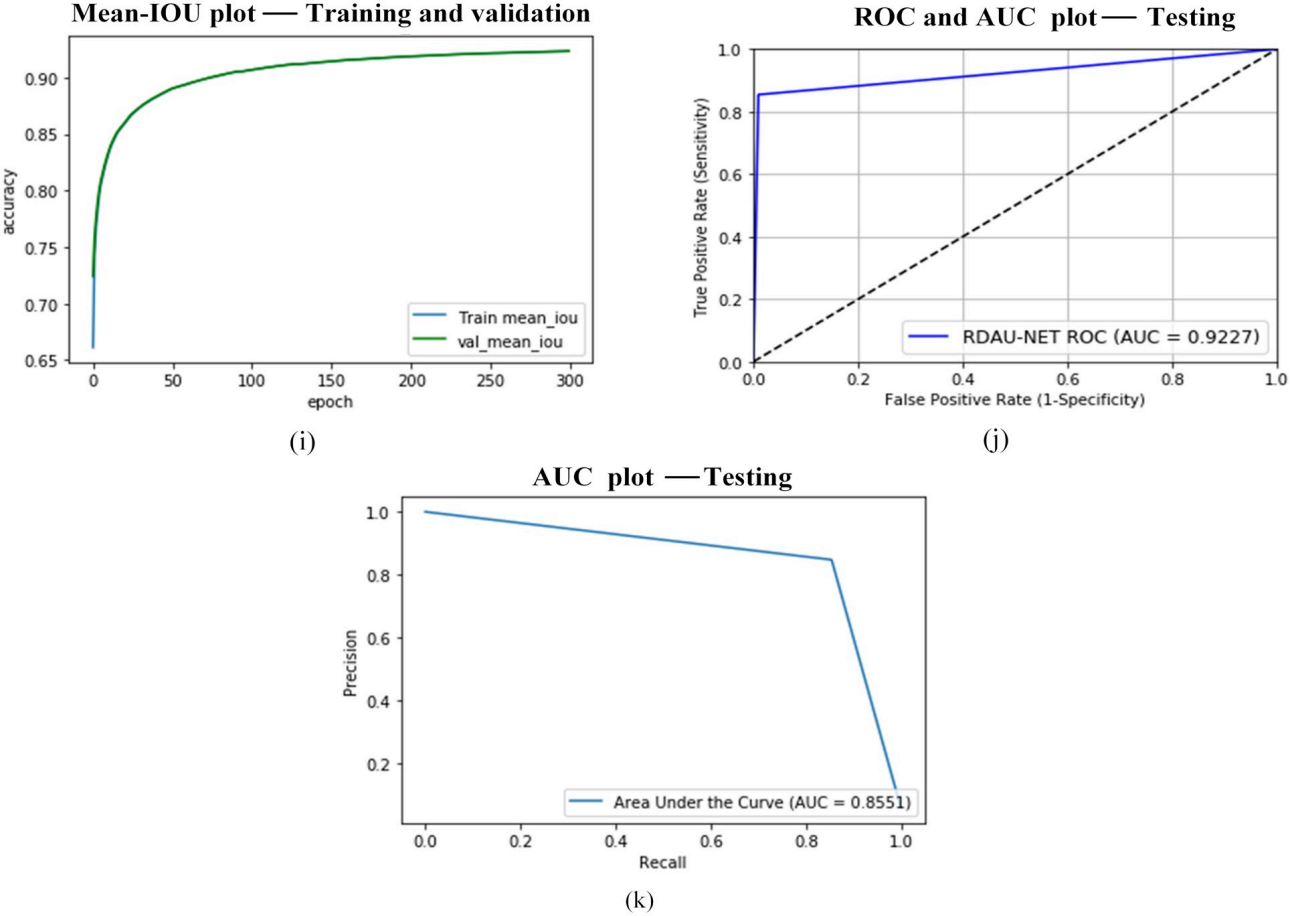
RDAU-NET performance indicators for training, validation, and testing. Here the plots (i) represent the performance metrics during training and validation and plots (j and k) specify the performance during testing: Fig 16(j) denotes ROC curve and AUC with respect to True Positive Rate and False Positive Rate and Fig 16(k) illustrate the AUC in relation to Precision and Recall.

Further, Figs 14–16 presents the performance curves obtained during the simulation the RDAU-NET during training, validation and testing process.

#### Quantitative comparison with other models

For the quantitative evaluation, a comparison was performed based on Eqs (9) to (12) between the segmented results of the proposed model and those obtained for the FCN8s, FCN16s, SegNet, U-Net, Residual U-Net, Squeeze U-Net, Dilated U-Net, RAU-NET, DAU-NET, RDU-NET, and RDAU-NET. The evaluation results are tabulated in Table 4.

**Table 4:** Quantitative segmentation results for different models based on the testing dataset.

Experimental Model	Loss	Acc	DC	Sen	Sp	F1	Pc	M-IOU	AUC
FCN8s	0.3676	0.9530	0.6323	0.7040	0.9729	0.6333	0.6085	0.7013	0.9500
FCN16s [33]	0.4507	0.9348	0.5492	0.7018	0.9528	0.5498	0.4842	0.6642	0.9147
SegNet [34]	0.1829	0.9752	0.8170	0.8395	0.9883	0.8171	0.8141	0.7914	0.9276
U-Net [22]	0.1795	0.9757	0.8204	0.8466	0.9891	0.8211	0.8185	0.7983	0.9269
Residual U-Net [35]	0.1746	0.9778	0.8253	0.8165	0.9930	0.8255	0.8670	0.7933	0.9181
Squeeze U-Net [36]	0.2077	0.9745	0.7922	0.7801	0.9909	0.7924	0.8425	0.7863	0.9301
Dilated U-Net [37]	0.1905	0.9740	0.8094	0.8433	0.9877	0.8098	0.8084	0.7784	0.9487
RAU-NET	0.1925	0.9768	0.8074	0.7847	0.9929	0.8081	0.8680	0.8023	0.9070
DAU-NET	0.1576	0.9781	0.8423	0.8392	0.9925	0.8431	0.8659	0.8035	0.9210
RDU-NET	0.1650	0.9784	0.8349	0.8107	0.9936	0.8356	0.8896	0.8087	0.9148
RDAU-NET	0.1530	0.9791	0.8469	0.8319	0.9934	0.8478	0.8858	0.8067	0.9227

The quantitative comparison presents the following conclusions from Table 4:

1. The segmentation performance of traditional U-Net is better than FCN8s, FCN16s, SegNet.
2. The segmentation results are comparatively better for Residual U-Net, Squeeze U-Net, and Dilated U-Net when compared with traditional U-Net. The improvement can be attributed to additional modules that are integrated into U-Net architecture.
3. In most of the evaluation parameters, the proposed RDAU-NET outperforms other models, and thus combing the three modules (Residual unit, Dilation unit, and Attention Gate) has provided accurate segmentation of lesions in BUS images.

## Conclusion

Though U-Net is a widely used model in medical image segmentation, it has not achieved the expected outcomes in BUS tumor segmentation. This is mainly due to the high noise, low contrast and weak boundary of ultrasound images. Therefore to achieve accurate segmentation, the model requires more powerful feature extraction and classification abilities. The new model, RDAU-NET proposed in the paper employed residual units dilated convolution and attention gate system top effectively segment the tumor region in BUS images. The experimental results show that the RDAU-NET model can accurately and efficiently segment the tumor region, and the final test results are superior to the traditional convolution neural network segmentation models, and hence has a great prospect for clinical application.
